# Worldwide host associations of the tick genus *Ixodes* suggest relationships based on environmental sharing rather than on co-phylogenetic events

**DOI:** 10.1186/s13071-022-05641-9

**Published:** 2023-02-21

**Authors:** Agustín Estrada-Peña, Alberto A. Guglielmone, Santiago Nava

**Affiliations:** 1 University of Zaragoza, Saragossa, Spain; 2 Instituto Nacional de Investigaciones Agropecuarias, Estación Experimental Agropecuaria Rafaela—Instituto de Investigación de la Cadena Láctea (INTA-Consejo de Investigaciones Científicas y Técnicas), Rafaela, Santa Fe Argentina

**Keywords:** *Ixodes* spp., Tick-host association, Environmental driving, Redundant networks, Cohesive relationships

## Abstract

**Background:**

This study aims to capture how ticks of the genus *Ixodes* gained their hosts using network constructs. We propose two alternative hypotheses, namely, an ecological background (ticks and hosts sharing environmentally available conditions) and a phylogenetic one, in which both partners co-evolved, adapting to existing environmental conditions after the association took place.

**Methods:**

We used network constructs linking all the known pairs of associations between each species and stage of ticks with families and orders of hosts. Faith’s phylogenetic diversity was used to evaluate the phylogenetic distance of the hosts of each species and changes occurring in the ontogenetic switch between consecutive stages of each species (or the extent of the changes in phylogenetic diversity of hosts for consecutive stages of the same species).

**Results:**

We report highly clustered associations among *Ixodes* ticks and hosts, supporting the influence of the ecological adaptation and coexistence, demonstrating a lack of strict tick-host coevolution in most cases, except for a few species. Keystone hosts do not exist in the relationships between *Ixodes* and vertebrates because of the high redundancy of the networks, further supporting an ecological relationship between both types of partners. The ontogenetic switch of hosts is high for species with enough data, which is another potential clue supporting the ecological hypothesis. Other results suggest that the networks displaying tick-host associations are different according to the biogeographical realms. Data for the Afrotropical region reveal a lack of extensive surveys, while results for the Australasian region are suggestive of a mass extinction of vertebrates. The Palearctic network is well developed, with many links demonstrating a highly modular set of relationships.

**Conclusions:**

With the obvious exceptions of *Ixodes* species restricted to one or a few hosts, the results point to an ecological adaptation. Even results on species linked to groups of ticks (such as *Ixodes uriae* and the pelagic birds or the bat-tick species) are suggestive of a previous action of environmental forces.

**Graphical Abstract:**

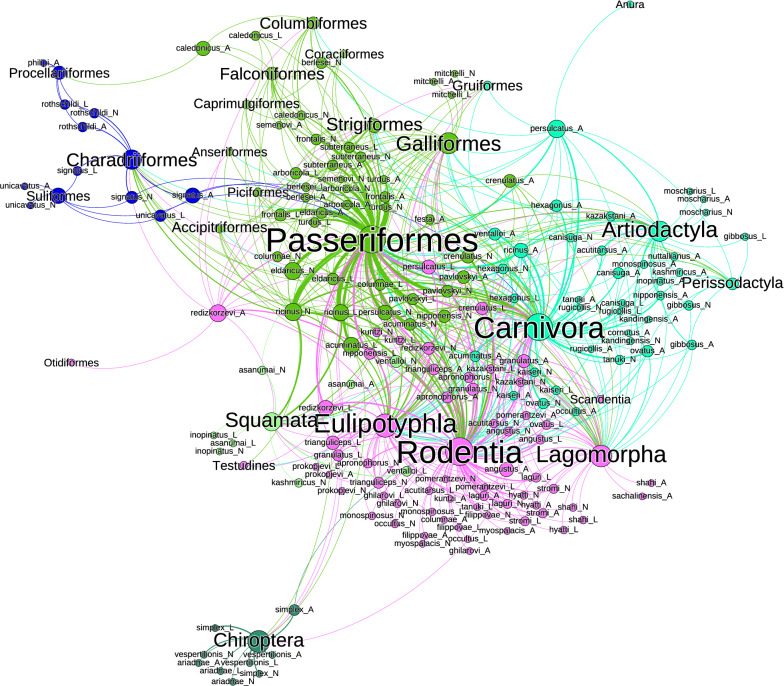

**Supplementary Information:**

The online version contains supplementary material available at 10.1186/s13071-022-05641-9.

## Background

The origin and evolution of ticks (Ixodoidea) is a topic that is still far from solved [1]. Advances have been reached resulting from multidisciplinary approaches, combining the sequencing of large portions of genome, molecular clocks, phylogenetic trees, comparative analyses of the proteins secreted in salivary glands (including physiological functions) and morphological traits. Pioneering views on the topic [2, 3] were supportive of a tick evolution linked to that of their hosts. These previous studies stated that almost 90% of the tick species described were “strict, or relatively strict” host specialists (as per categories in [3]). However, this view linking the speciation of ticks as linked to the hosts has been criticized. The compilation of available data from the literature produced positive correlations between the assigned host specificity and the sampling effort of a given species [4]. Different meta-analyses clearly demonstrated that ecological specificity is more important than the association with a certain group of hosts. This finding translates into the importance of adaptation to a particular environment where the nonparasitic phase is developed, being more relevant than the parasite-host coevolution [4–7].

The evolutionary origin of ticks is still debated, mainly because of the limited fossil record. The fossil evidence and molecular clock analyses suggest that the present-day genera *Ixodes* and *Amblyomma*, representatives of Prostriata and Metastriata, respectively, were already differentiated at least by the Cretaceous period [8, 9]. An origin of ticks in Gondwana is assumed using the most conservative dates [10], dating the event around the late Carboniferous (∼320 MYA) or Early Permian (∼290 MYA), together with the evolution of a blood-feeding lifestyle on early therapsid, diapsid or synapsid hosts [8, 11]. Data also demonstrated that Australasian *Bothriocotron* do not have a basal position and that non-Australian *Ixodes* are basal to Australian *Ixodes* [12, 13], suggesting that the *Ixodes* lineage originated outside Australia. However, Australian and non-Australian *Ixodes* result as sister groups in these phylogenies [13]. A possible origin of Ixodidae in the Antarctic region with further dispersal has been elaborated by Mans et al. [11]. These authors argued that an impressive fauna populated the region during the Permian and Triassic, and basal lineages that ended up in Australia would have suffered mass extinction events during its glaciation [14]. As stated by Mans et al. [10], “this may explain the relatively small numbers of extant species in the Australian tick lineages, as well as their limited distributions.”

A deeper understanding of how ticks “gained” their hosts would have both ecological and epidemiological implications, since some vertebrates act as competent reservoirs of prominent pathogens, affecting human and animal health. It seems that ticks gained their hosts, from their primitive conditions of nonpredatory, nonparasitic scavengers, using the “available” therapsids at the time. While phylogenetically connected, these primitive hosts were far from the current tick-host associations, which are based on a scheme formed by birds and mammals of several orders, and/or reptiles/snakes, probably originated in the Cenozoic after the extinction of dinosaurs except Aves. One exception to this common background of hosts are the marsupials, believed to have evolved in the current North America, approximately 90 MYA, expanding into South America and the Pacific rim of Asia. Marsupials are unknown in the fossil record of Asia and Africa. It is accepted that modern marsupials arose in the late Cretaceous [15]; it has been noticed that several species of ticks are associated with marsupials in one or several stages of their life cycle [16, 17]. An additional factor is the development of new associations of ticks with exotic domestic and wild animals, introduced recently into an area (hundreds or tens of years), which could obliterate some relationships of a tick species with its primeval wild host(s), leading to new parasite-host associations.

On the other hand, specific environmental conditions (also known as the “abiotic niche”) are essential for ticks, because they expend their off-host cycle (approximately 95% of their total life cycle) questing for a host or molting in the vegetation; this niche is shaped by temperature, humidity, and other variables that may affect tick survival and development. Ticks, like any other organism, track their abiotic niche [18] but the hosts are the biotic part of the tick’s niche. Thus, a basic question persists: did the primitive ticks adapt to portions of the abiotic niche, feeding on the existing vertebrates at such niches, or did ticks phylogenetically coevolve with vertebrates, ticks exploiting only the niches colonized by these vertebrates? In the former option, ticks would feed on lineages of vertebrates living in gradients of the abiotic niche. In the latter, ticks would be restricted to a clade of vertebrates; the speciation of clades of vertebrates could involve the exploitation of new niches, carrying ticks with them. The terms “generalist” (a catholic feeder) or “specialist” ticks (feeding on a narrow range of vertebrates) do not solve the question: a specialist tick could be restricted by either host-derived phylogenetic constraints or by the climate gradients, avoiding further spread. The current host-parasite relationships are blurred by extinctions, long-term trends of climate, migration phenomena, movements of land masses and speciation events produced under unknown forces.

We aimed to apply methods gathered from ecological studies, applied to the ticks of the genus *Ixodes*, as a proof-of-concept for exploring tick-hosts relationships. The genus includes 277 species currently accepted as valid and is distributed worldwide (including small islands in the Arctic and Antarctica). Using a compilation of the known host range of *Ixodes* spp., we wanted to analyze these relationships and explore clues to demonstrate how this large tick genus gained its hosts. We hypothesized that the associations between ticks and hosts are highly clustered and redundant. The first term means that these associations form independent clusters. In this context, “redundancy” means the existence of several or many phylogenetically unrelated hosts supporting the same tick species. The latter concept has been slightly explored before [19] and is believed to result in resilient networks of ticks and hosts, in which the removal of some hosts would not result in a large perturbation of the system. This would be demonstrative of a lack of strict tick-host coevolution in most cases. Further aims of this study were (i) networks displaying tick-host relationships are different at each biogeographical realm, as an echoing hallmark of the original associations evolved over time, (ii) some indexes of these networks, such as nestedness and modularity, are important parameters defining the resilience of relationships among partners, and (iii) keystone hosts do not exist in the relationships between *Ixodes* and vertebrates because of the high redundancy of the networks (but in a few species). The latter would support an environmental dependence. We were also interested in the ontogenetic switch of hosts as another potential clue supporting either hypothesis. *Ixodes* spp. have three stages feeding on different hosts. There are species of *Ixodes* that use groups of hosts that are widely separated in the phylogenetic tree, at each stage of feeding; other species are restricted to one or a few species of vertebrates in the three stages. The extent of an ontogenetic switch in the genus would support an environmentally driven adaptation of these ticks to hosts.

## Methods

### Data collection

All the data used in this study on the associations of *Ixodes* spp. and vertebrates have been compiled by AAG and SN and almost completely published [20]. Additional data not included in the reference work were also compiled by these coauthors after the year 2020: they included corrections and new tick-vertebrate associations that appeared in the literature after the year 2019 or were newly located in unnoticed references. Vertebrates are used in this study at the taxonomic level of families and orders. Attempts to systematize the associations at generic or specific levels in vertebrates are prone to data incompleteness, misidentification, over-representions of some species or mistakes in the reporting of the specific names of the hosts.

### Phylogenetic tree of hosts of the genus *Ixodes*

Several indexes calculated in this study rely on a phylogenetic tree of the vertebrates, which we chose to be based at the family level. We used a synthetic tree obtained from the Open Tree of Life (OTL), accessing its application programming interface using the library “rOTL” [21] for the R programming environment [22]. In short, we prepared the list of families of vertebrates on which each species of *Ixodes* has been reported, queried OTL and obtained a non-ultrametric tree of these families. We obtained the length of each branch of the tree for further calculations using commands in R. The complete network of tick-host associations and the phylogenetic tree in Nexus format are provided as Additional file 1.

### Phylogenetic diversity of the hosts of each tick species

The explicit comparison between the phylogenetic trees of both ticks and hosts could provide interesting data about coevolution events and even with details about the date these events could occur. However, it would be necessary to have a phylogenetic tree including every species of tick, allowing plausible comparisons between the two trees (vertebrates and ticks). We aimed to prepare that tree for ticks based on genetic sequences available in GenBank. We however realized that some sequences are inadequately labeled, or they belong to misidentified ticks, as noticed after alignment. Furthermore, the same genes have not been systematically used in previous research about this tick genus; additionally, some tick species are over-represented while sequences for others are not available. We assessed that approximately 20% of *Ixodes* spp. were represented in GenBank, preventing this approach, in favor of other strategies.

One of the main indices in the evaluation of the host associations in the genus *Ixodes* is the calculation of the phylogenetic diversity (PD) of the hosts reported for each species of tick, using Faith’s index of phylogenetic diversity [23]. The PD is defined as the sum of the lengths of all those branches on the tree that span the members of the set of hosts for each stage and species of tick. The branch lengths on the tree are informative because they count the relative number of new features arising along that part of the tree. This is practical in our context, because we deal with families of vertebrates (consisting of genera and species) in which the number of species may be very different and for which we may not have adequate information. The calculation of PD was complemented by the number of parasitized families by each tick species (a higher number of parasitized families may explain a higher PD, but also a few, largely unrelated hosts may also explain high values of PD). All the computations regarding PD were performed separately for each realm and known stage(s) of each species.

### Network approach and its indices

Ecological networks of any kind, such as food webs or plants plus pollinators, may be highly structured [24]; such structuring demonstrates that species do not interact randomly with each other [25, 26]. Considering the nature of the data (parasites and hosts), we chose a graph construct as the most appropriate method to handle and represent data and to extract ecological or phylogenetic meanings behind these relationships. A graph (or network) is a set of nodes (the organisms, e.g. ticks and vertebrates) that interact among them through links. Both *Order* and *Size* measure the number of nodes and number of links, respectively. Noninteracting organisms have no links between them. Interacting organisms are always pairs of nodes (i.e. the tick species “a” has been recorded on the vertebrate “b”). We chose to express the data as a bipartite network whose nodes are divided into two sets X (ticks) and Y (hosts), and only connections between two nodes in different sets are allowed (a tick cannot parasitize another tick, and a vertebrate cannot host another vertebrate). The number of times each tick has been cited in a family of vertebrates is the weight of the link between the nodes of tick and vertebrate. We also used orders of vertebrates to summarize the data, mainly aiming to improve the visualization of charts.

Other than analyzing the complete network formed by every species of ticks we used the known distribution of each tick according to biogeographical realms (Afrotropical, Oriental, Australasian, Nearctic and Neotropical) to prepare “partial” networks involving only records from these regions. Some ticks share two or more realms and have been included in both. A few species of ticks have a worldwide range and were included only in the complete network. The aim for different networks is to follow a coherent overview of tick-vertebrate relationships along biogeographical regions.

We used Gephi 0.92 for network calculations (http://www.gephi.org, last accessed February 2022). We used several indices for characterizing each network, namely the weighted degree, betweenness centrality and modularity. In weighted networks the weighted degree is calculated as the sum of weights assigned to the node's direct connections and represents the node strength, adjusted by the weight of each edge. It is based on the weights of links and not only on the number of links. Centrality measures, such as betweenness centrality, can be used to detect which hosts play a key role in a network. The algorithm for betweenness centrality calculates the shortest paths between all pairs of nodes in a graph, describing the importance of a node as “connector.” For example, if many species use Rodentia as hosts, then Rodentia will have a high betweenness, and its removal most probably promotes a leaking of the network in separated modules. Modularity is a measure of clustering in subsets of interacting nodes. It detects groups of nodes that interact statistically more frequently among them than with the rest of nodes. Modularity was calculated using the Louvaine algorithm, as included in Gephi 0.92. A parasite-host network with high modularity is not synonymous with high fragility of the interactions, but of tight relationships among nodes, well separated from the relationships of other ticks and hosts. Other than these indexes, both *order* and *size* indicate the number of interacting organisms and the resulting links among them, respectively.

We calculated other indices available in the package “bipartite” [27] for R. These indices are *links per species* (the sum of links divided by the number of species of ticks), *number of compartments* (subsets of the network that are not connected to another compartment) and *nestedness*, a measure of structure in an ecological system, usually applied to interactions among species such as hosts-parasites. A network is said to be nested when the clusters that have a few items in them are a subset of the items of elements with more items. We calculated the Nestedness Overlap and Decreasing Fill (*NODF*) as the index for the nestedness of the matrix, in which high values indicate nestedness [28]. The interaction diversity (ticks on vertebrates) was measured by the *Fisher’s alpha* [29, 30]. *Functional complementarity* and *partner diversity* are two indices that measure how the species of ticks “share” or not similar groups of hosts. The former is measured as the amount of functional space shared by the community, and the second is the weighted mean Shannon diversity of the number of interactions of the species of ticks. Last, the *extinction slope* measures the slope of the effects of hosts’ removal into the “extinction” sequence of the tick species; the method is also a measure of robustness of the system to the losses of hosts. The method is based on the fact that if a given fraction of hosts is eliminated, several species of ticks that depend on these interactions could become “extinct” [31].

### Ontogenetic switch of hosts in each stage of the ticks

*Ixodes* is a tick genus whose three stages feed on three different hosts. They molt between consecutive stages, commonly on the ground, but also in nests or shelters of the hosts. For example, it is relatively common that the immature stages of a tick feed on small animals; adult ticks of some species need a copious blood meal and are commonly reported on large animals. However, other species may use small animals for feeding even as adults. We aimed to examine how the different stages of *Ixodes* switch the hosts, in what is called the “ontogenetic shift.” To capture a hypothetical evolutionary linkage of ticks to hosts, it is necessary to know how every stage is restricted to given lineage(s) of hosts. Such restriction to hosts among stages could reveal a phylogenetic clustering to a group of vertebrates and could be detected by a low variability of phylogenetic distance among hosts of each stage. If tick stages “switch” among hosts, then a lack of evolutionary linkage between ticks and vertebrates should be suspected, which should be proportional to a higher phylogenetic diversity among the hosts of each stage.

Calculations were based on Faith’s phylogenetic diversity on the phylogenetic tree of the families of hosts built as explained before, computing the rate of change of PD of the hosts reported for two consecutive stages. A value near zero means that both stages of the tick share similar hosts that are phylogenetically close. A large value between consecutive feeding stages is an indication of switching. This evaluation was performed separately for the tick-vertebrate association in each biogeographical realm, and always for the three consecutive life cycle stages; the Oriental region was not included because the hosts for each stage are known for only six species of *Ixodes* in that region. We also calculated the phylogenetic diversity of the hosts of each species of tick together with the probability of co-occurrence of ticks on different groups of vertebrates calculating the observed and expected frequencies of co-occurrence between each pair of species. In the latter, when a species pair is not classified as positive or negative, the species pair can be truly randomly distributed, or they can be unclassifiable because of low statistical power.

## Results

### The network of *Ixodes* spp. is a highly modular, loosely nested construct

**Fig. 1:**
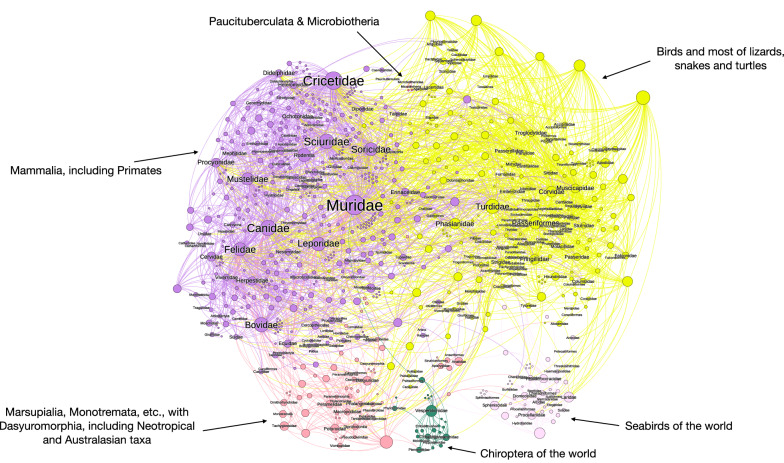
Complete network of *Ixodes* spp., families and orders of hosts, arranged using the Force Atlas 2 algorithm. Circles (nodes) are taxa, but only hosts carry a label for improved readability (the same figure with the complete set of labels is available as Additional file 1, allowing on-screen zoom). The lines (links) between pairs of nodes are relationships between ticks and hosts (i.e. a species of tick reported on a family of hosts). The width of the line is proportional to the number of co-occurring events, but the length results only from the algorithm producing the chart. Colors are clusters or groups of ticks and hosts that interact more frequently among them than with other taxa. Labels indicate the main groups of vertebrates

Figure 1 displays the network that includes all the species of *Ixodes* and their hosts. It includes labels only for the families and orders of vertebrates to improve readability. Additional file 2 includes the same figure with all the labels, allowing on-screen zoom. The construct resolves the relationships as six clusters with a variable number of taxa: two large clusters are formed by Aves (excluding pelagic species) and the Eutheria (excluding bats); lizards, snakes and turtles are included in the cluster of Aves. The taxa in the cluster hosting Rodentia, Carnivora and Artiodactyla are linked with the previous clusters because the different stages of several *Ixodes* species can feed on different clusters of hosts (see below, ontogenetic switch). For instance, Cricetidae, Muridae and Sciuridae may be hosts of immature stages of *Ixodes* spp. associated with Marsupialia in both adults and immature stages. In the same way, the importance of Passeriformes in supporting many *Ixodes* spp. is overwhelming, and the order is also used by ticks feeding also on Eutheria (mainly Rodentia). The importance of Passeriformes is anecdotical for the *Ixodes* spp. currently known in the Australasian region.

Pelagic birds (e.g. Charadriiformes and Procelariiformes) together with Sphenisciformes form a remarkable cluster separated from the rest because it has tick species that use these hosts at every stage (see Fig. 1 and Additional file 2). This means a separate and unique set of relationships unrelated to other ticks. However, the cluster is not separated from the main network because several species of generalist ticks have been recorded on pelagic birds and other Aves (most notably the three stages of the species in the *Ixodes auritulus* group). The bats of the world form a similarly well separated cluster, together with a few species of *Ixodes* that seem to be specialists of the group of hosts. As before, the cluster is not separated from the main network because some generalist ticks (belonging to the clusters of Aves and Mammalia) have been reported on bats. The Marsupialia, Monotremata and Dasyuromorphia of both Neotropical and Australasian realms resulted in another cluster separated from the mainstream of relationships detected for *Ixodes*. Interestingly, the Struthioniformes (Apterygidae) are included in the same group. Paucituberculata and Microbiotheria form the smallest cluster of the network.

The complete network of *Ixodes* spp. and their hosts has 3.35 links per tick species (Table 1) but is only slightly nested (NODF = 14.42, in the scale 0–100). This promotes a higher modularity (i.e. more clusters). The indexes above are indicative of a very solid construct because of the high redundancy (Fisher alpha = 1436.98) since *Ixodes* spp. use a total of 222 families of hosts. The high functional complementarity of the complete network (963.89) is a consequence of the large number of links mainly to Eutheria and Aves, enhancing redundancy with the connections between the two main groups of vertebrates. Such large number of links with these two groups of hosts produces a relatively high index of partner diversity (2.38).

**Table 1:** Main indexes of the complete and separate networks of *Ixodes* spp. and their reported hosts

	Complete	Neotropical	Nearctic	Afrotropical	Palearctic	Australasian	Oriental
Order	864	124	88	144	189	121	100
Size	3123	185	170	268	572	236	179
Links per species	3.35	1.46	3.75	2.19	4.52	2.09	1.49
Number of clusters	6	8	5	7	6	8	8
NODF	14.42	13.05	38.05	18.14	30.81	18.70	21.97
Fisher alpha	1436.98	679.77	1363.26	1191.25	2115.51	693.41	512.91
Number of families of hosts	222	54	76	69	93	76	34
Functional complementarity	963.89	107.34	199.54	177.82	324.79	141.47	41.06
Partner diversity	2.38	1.23	2.47	1.69	2.61	2.06	1.52
Extinction slope	2.80	2.16	3.19	2.37	3.95	2.40	1.83

Data for both the complete network (i.e. including every known species until December 2021) and the networks for the different biogeographical realms are included. The indexes are defined in the Methods section

### Relationships between *Ixodes* and vertebrates differ along biogeographical realms

**Fig. 2:**
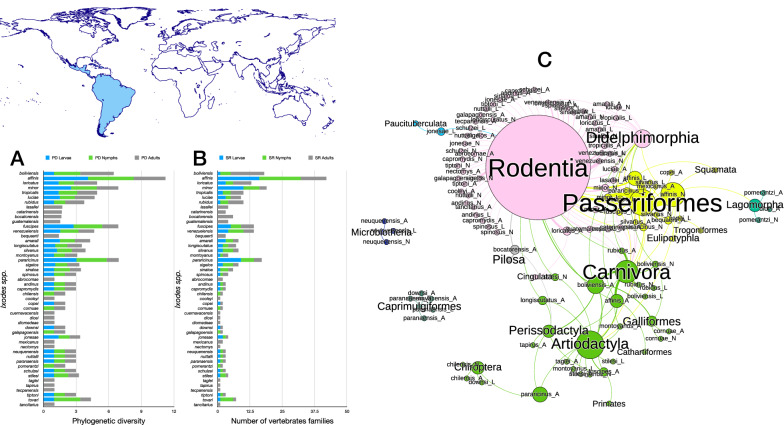
Complete network of *Ixodes* spp., families and orders of hosts for the Neotropical region. Included are data for the phylogenetic diversity calculated according to Faith’s index (**A**), the richness of families of vertebrates in which the ticks have been reported (**B**) and the network of relationships among organisms (**C**). Species of ticks in A and B are sorted according to the value of the adult stage, in decreasing order

**Fig. 3:**
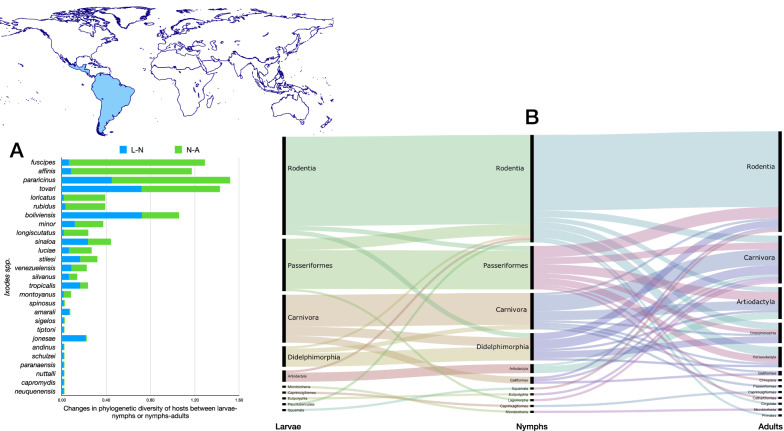
Ontogenetic switch of host for the species of *Ixodes* recorded in the Neotropical region. Bars in A indicate the change of the value of phylogenetic diversity in either the molt larva-nymph (L-N) or nymph-adult (N-A). A value of 0 means “no change” and a value of 1 means “twice value.” Species are sorted in decreasing order after the value for the nymph-adult molt. An alluvial chart is displayed in B, showing the “flow” of records of hosts for the consecutive stages in the tick’s life cycle. The width of the bands is proportional to the percent of records of ticks that switch families of vertebrates as hosts between two consecutive stages; this chart is intended as visual information on the ontogenetic switch of hosts

**Fig. 4:**
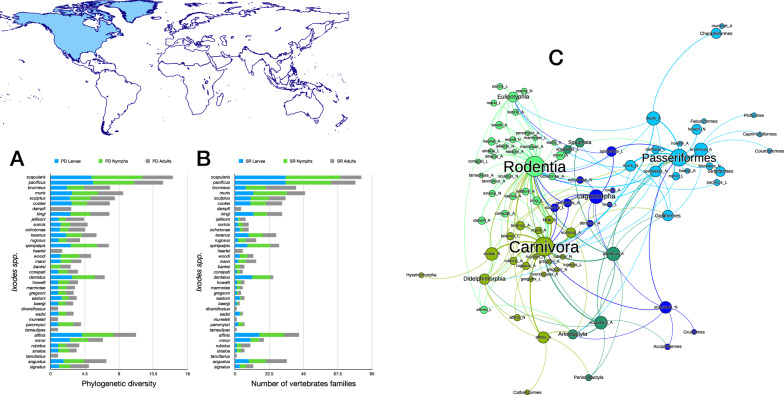
Complete network of *Ixodes* spp., families and orders of hosts for the Neartic region. Included are data for the phylogenetic diversity calculated according to Faith’s index (**A**), the richness of families of vertebrates in which the ticks have been reported (**B**) and the network of relationships among organisms (**C**). Species of ticks in A and B are sorted according to the value of the adult stage, in decreasing order

**Fig. 5:**
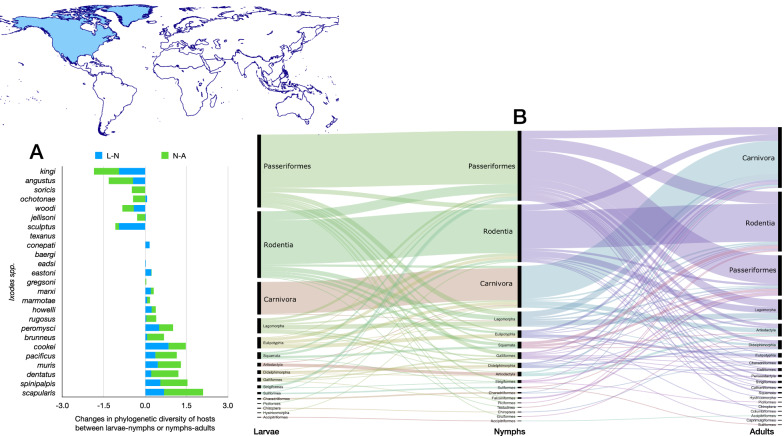
Ontogenetic switch of host for the species of *Ixodes* recorded in the Neotropical region. Bars in A indicate the change of the value of phylogenetic diversity in either the molt larva-nymph (L-N) or nymph-adult (N-A). A value of 0 means “no change” and a value of 1 means “twice value.” Species are sorted in decreasing order after the value for the nymph-adult molt. Note that some species have negative values, denoting a decrease of phylogenetic diversity between two consecutive stages. An alluvial chart is displayed in B, showing the “flow” of records of hosts for the consecutive stages in the tick’s life cycle. The width of the bands is proportional to the percent of records of ticks that switch families of vertebrates as hosts between two consecutive stages; this chart is intended as visual information on the ontogenetic switch of hosts

**Fig. 6:**
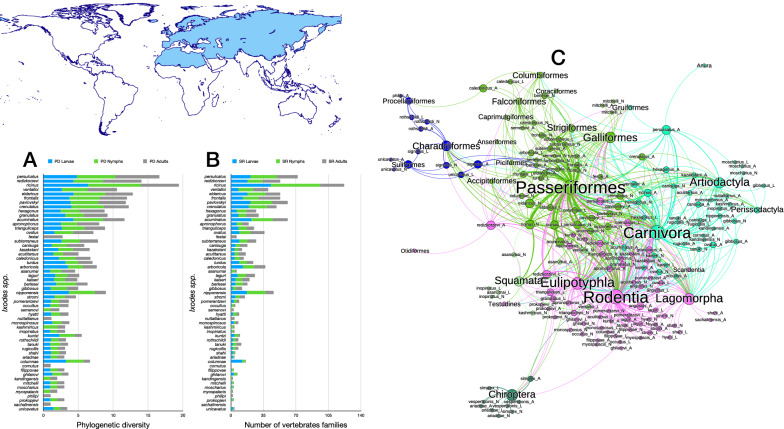
Complete network of *Ixodes* spp., families and orders of hosts for the Palearctic region. Included are data for the phylogenetic diversity calculated according to Faith’s index (**A**), the richness of families of vertebrates in which the ticks have been reported (**B**) and the network of relationships among organisms (**C**). Species of ticks in A and B are sorted according to the value of the adult stage, in decreasing order

**Fig. 7:**
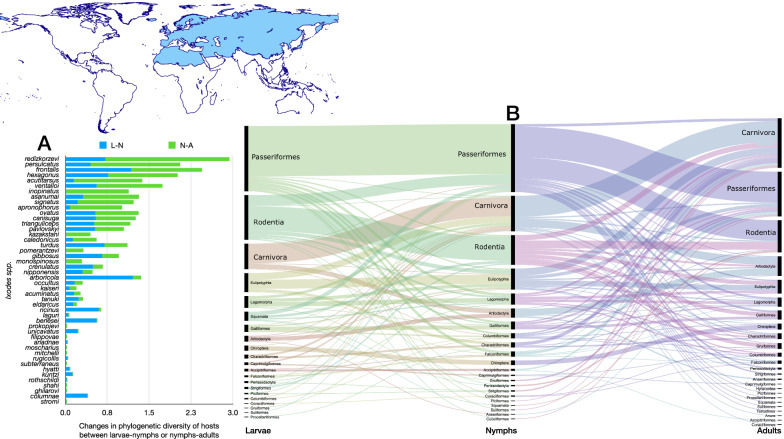
Ontogenetic switch of host for the species of *Ixodes* recorded in the Neotropical region. Bars in A indicate the change of the value of phylogenetic diversity in either the molt larva-nymph (L-N) or nymph-adult (N-A). A value of 0 means “no change” and a value of 1 means “twice value.” Species are sorted in decreasing order after its value for the nymph-adult molt. Note that some species have negative values, denoting a decrease of phylogenetic diversity between two consecutive stages. An alluvial chart is displayed in B, showing the “flow” of records of hosts for the consecutive stages in the tick’s life cycle. The width of the bands is proportional to the percent of records of ticks that switch families of vertebrates as hosts between two consecutive stages; this chart is intended as visual information on the ontogenetic switch of hosts

**Fig. 8:**
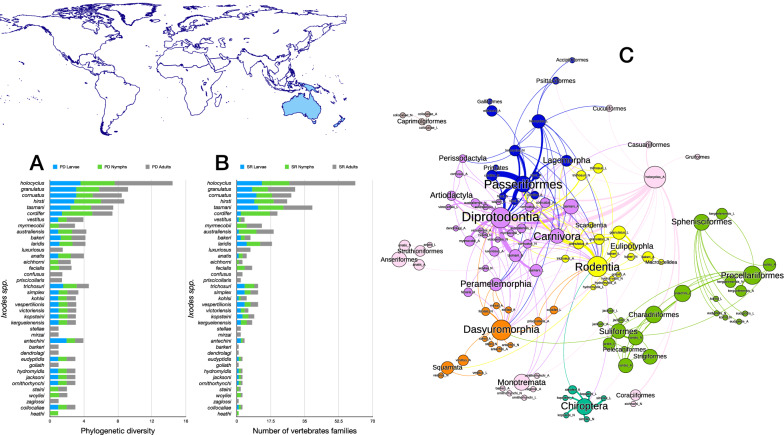
Complete network of *Ixodes* spp., families and orders of hosts for the Australasian region. Included are data for the phylogenetic diversity calculated according to Faith’s index (**A**), the richness of families of vertebrate in which the ticks have been reported (**B**) and the network of relationships among organisms (**C**). Species of ticks in A and B are sorted according to the value of the adult stage, in decreasing order

**Fig. 9:**
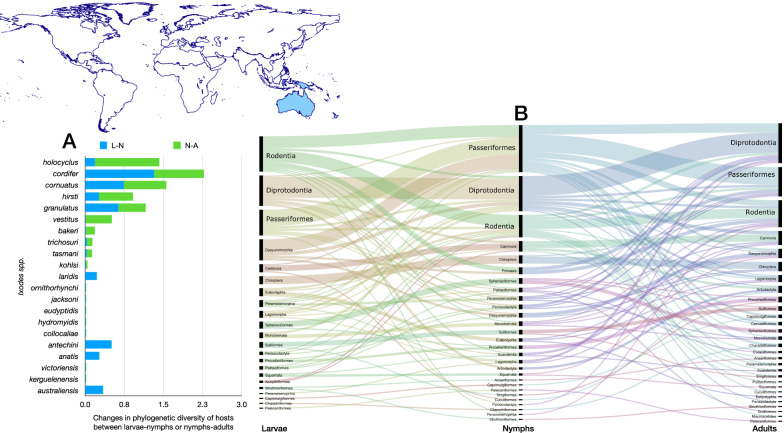
Ontogenetic switch of host for the species of *Ixodes* recorded in the Neotropical region. Bars in A indicate the change of the value of phylogenetic diversity in either the molt larva-nymph (L-N) or nymph-adult (N-A). A value of 0 means “no change” and a value of 1 means “twice value.” Species are sorted in decreasing order after the value for the nymph-adult molt. Note that some species have negative values, denoting a decrease of phylogenetic diversity between two consecutive stages. An alluvial chart is displayed in B, showing the “flow” of records of hosts for the consecutive stages in the tick’s life cycle. The width of the bands is proportional to the percent of records of ticks that switch families of vertebrates as hosts between two consecutive stages; this chart is intended as visual information on the ontogenetic switch of hosts

**Fig. 10:**
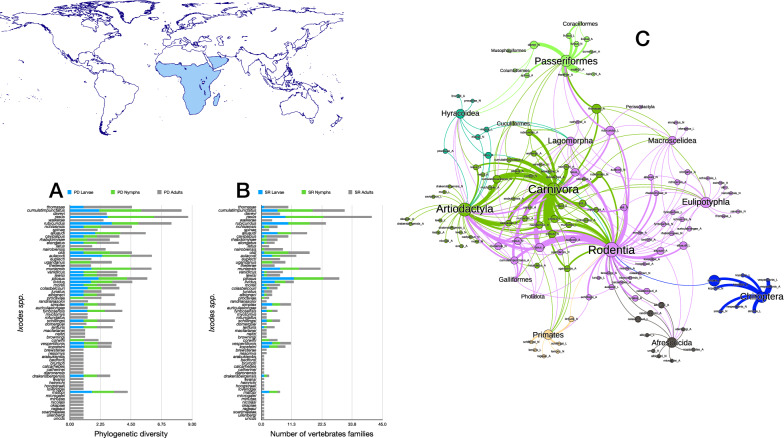
Complete network of *Ixodes* spp., families and orders of hosts for the Afrotropical region. Included are data for the phylogenetic diversity calculated according to Faith’s index (**A**), the richness of families of vertebrates in which the ticks have been reported (**B**) and the network of relationships among organisms (**C**). Species of ticks in A and B are sorted according to the value of the adult stage in decreasing order

**Fig. 11:**
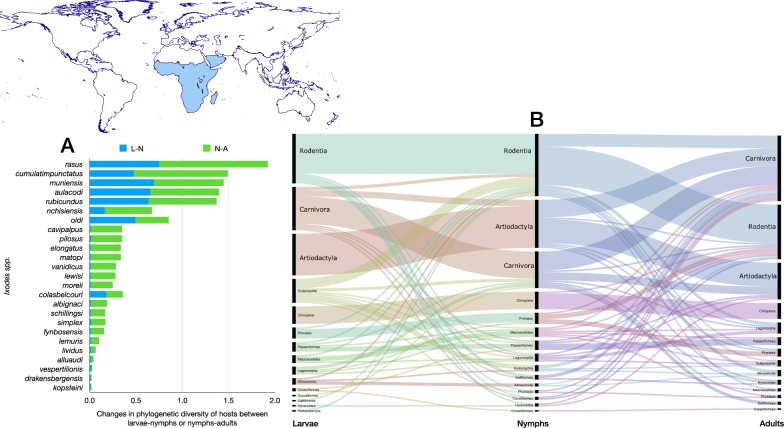
Ontogenetic switch of host for the species of *Ixodes* recorded in the Neotropical region. Bars in A indicate the change of the value of phylogenetic diversity in either the molt larva-nymph (L-N) or nymph-adult (N-A). A value of 0 means “no change” and a value of 1 means “twice value.” Species are sorted in decreasing order after the value for the nymph-adult molt. Note that some species have negative values, denoting a decrease of phylogenetic diversity between two consecutive stages. An alluvial chart is displayed in B, showing the “flow” of records of hosts for the consecutive stages in the tick’s life cycle. The width of the bands is proportional to the percent of records of ticks that switch families of vertebrates as hosts between two consecutive stages; this chart is intended as visual information on the ontogenetic switch of hosts

We split the results of the complete network into five different networks representing the biogeographical realms of the world (see Figs. 2, 3, 4, 5, 6, 7, 8, 9, 10, 11) displaying the results. The network of the ticks in each realm is also included in the figures. The data for the Oriental region must be taken with caution, because there are records of hosts for the three stages of only six species (*Ixodes spinicoxalis, Ixodes ceylonensis, Ixodes petauristae, Ixodes himalayensis* and *Ixodes werneri*, in decreasing order of phylogenetic diversity of adults), together with *Ixodes laysanensis* present in the Pacific rim.

#### Neotropical region

Four species of *Ixodes* ticks in the Neotropical region (Figs. 2, 3) have the highest phylogenetic diversity values for all three stages, namely *Ixodes affinis, Ixodes minor, Ixodes fuscipes* and *Ixodes pararicinus*. Of these, the highest phylogenetic diversity is found for the larvae of *I. affinis* (which is considered a complex of species). Such high phylogenetic diversity is not paralleled by the number of families of vertebrates (*n* = 38) recorded as hosts of the species. This network has the highest modularity and lowest nestedness, redundancy, functional complementarity and host diversity. Interestingly, the network shows several clusters separated from the main group of species (i.e. Caprimulgiformes and Microbiotheria). The Paucituberculata are linked to the main body of the network by only one species and stage (larvae of *Ixodes jonesae*). Similar findings apply for Chiroptera and the adults of *I. pararicinus*. The largest clusters of the network are those formed by Rodentia and Passeriformes, with the former observing a large share of hosts also with Didelphimorphia (Fig. 2).

The ontogenetic switch of hosts is clear in many species except in those associated with Rodentia. The three stages of approximately 60% of the Neotropical species are associated with Rodentia. Those whose larvae and nymphs are associated with Passeriformes or Carnivora as larvae or nymphs are later associated with a large variety of host orders (Fig. 3).

#### Nearctic region

Nearctic region together with species found also in the Netropics—*I. affinis*—and species recorded also in the Palearctic—*Ixodes angustus* and *Ixodes signatus* (Figs. 4, 5). The network of *Ixodes* ticks in the Nearctic has a high number of links per species, only two clusters, the highest nestedness, the highest partner diversity and a solid resilience to extinction (i.e. removal of hosts, measured as the extinction slope). A small number of clusters means a tightly aggregated network (confirmed by the large nestedness) with redundancy of links (the same species and stage of ticks feed on vertebrates of several families). This results in a very simple network (Fig. 4), in which Rodentia, Passeriformes and Carnivora are the main hosts. Both *Ixodes scapularis* and *Ixodes pacificus* have the highest phylogenetic diversity of hosts in the realm. Up to 10 species found in the Nearctic have a PD value > 4. Both *I. scapularis* and *I. pacificus* have been recorded in > 80 families of vertebrates; the next species in the rank is *Ixodes muris*, with 46 families; the rest of Nearctic *Ixodes* spp. were recorded in < 25 families of vertebrates each.

The ontogenetic switch of hosts is similar to that explained for the Neotropics. Passeriformes, Rodentia and Carnivora are the main orders of hosts linked to larval and nymphal stages. Interestingly, most of these relationships remain for the adult stage in the Nearctic, a feature not found in other realms. The panorama in Fig. 5 suggests a large ontogenetic switch from larvae feeding on Rodentia and Passeriformes to nymphs and adults on other orders and then back as adults to the same groups of vertebrates as larvae. Simultaneously, nymphs that remained linked to Passeriformes-Rodentia use other orders as adults. The highest values of ontogenetic switching between nymphs and adults are recorded for *Ixodes scapularis*, *Ixodes pacificus*, *Ixodes muris*, *Ixodes brunneus* and *Ixodes sculptus*.

#### Palearctic region

The network of *Ixodes* ticks in the Palearctic (Figs. 6, 7) shares several important features with the network built for Nearctic species. Without question, it is more complex in terms of the number of species of ticks and orders of hosts (Table 1), the most diverse (4.52 links to hosts per tick species) and the highly nested (NODF = 30.81). The Palearctic *Ixodes* spp. used 93 families of hosts, resulting in the highest functional complementarity and partner diversity, with the highest resilience to extinction after consecutive removal of hosts (extinction slope = 3.95). Some interesting features of the Palearctic *Ixodes* are the use of many orders of Aves, together with Rodentia, Lagomorpha, Eulipotyphla and Carnivora and Artiodactyla. The species parasitic on seabirds and on Chiroptera form associations that are clearly separated from the main body of the network. However, these two “subnetworks” remain attached to the main core by several species/stages linked to either Passeriformes or Rodentia (Fig. 6).

Most species of the group *Ixodes ricinus* have the highest phylogenetic diversity. In this group, maximum values have been recorded for *Ixodes persulcatus*, *Ixodes redikorzevi* (treated here as an independent species, not as synonym of *Ixodes acuminatus*), *Ixodes ricinus*, *Ixodes ventalloi* and *Ixodes eldaricus*. Without question, *I. ricinus* is the species with the largest value of phylogenetic diversity and of host richness for immatures. Both larvae and nymphs were collected from 96 families of vertebrates, resulting in a phylogenetic diversity of > 7.5 (nymphs) and 6.3 (larvae). Closer species in the same realm (such as *I. persulcatus*) or in others (such as *I. scapularis*) do not have such high values, even if the survey effort is expected to be similar.

Ontogenetic switch in the Palearctic *Ixodes* spp. has peculiar features. As in the Neotropic and Nearctic, the main hosts for all stages are Passeriformes, Rodentia and Carnivora, with several species recorded on a few occasions on other orders. The most significant feature is the high number of species whose adults feed on Passeriformes, with a major nymph-adult switch from Passeriformes to Passeriformes + Rodentia (Fig. 7). The Palearctic *Ixodes* have the highest rates of phylogenetic diversity, comparing either between larva-nymph or nymph-adult. Integrating together the network-derived indices with the chart on ontogenetic switch, it seems that these high values are derived not from a complete switch of hosts (new hosts replacing others) but because the high plasticity of the ticks results in many groups of hosts used.

#### Australasian region

The network of Australian *Ixodes* spp. (Figs. 8, 9) and their hosts displays the highest number of disconnected clusters, the lowest functional diversity and the lowest functional complementarity. Important features are the high number of clusters and the lack of connection with the core of the network. The Australasian *Ixodes* spp. use as many as 76 families of hosts, but they belong to very different groups and are not connected among them (i.e. there is no redundancy). Species parasitic on seabirds (Suliformes, Charadriiformes, Pelecaniformes and Procellariformes) have links with Strigiformes and Sphenisciformes. Without the records of *Ixodes kerguelenensis* or *Ixodes kohlsi*, the ticks on penguins would be separated from those of seabirds. Similar findings are observed in the cluster of Chiroptera: only the record of *Ixodes simplex* on Rodentia links the Australasian bats with the core of the network. These are probably casual findings or misidentifications impossible to recheck. Monotremata and Diprotodontia also have a few links relating both groups of vertebrates regarding their *Ixodes* ticks. However, Dasyuromorphia is linked to Peramelemorphia and, to a lesser degree, to Diprotodontia. In other words, the endemic fauna of Australia exhibits a low degree of sharing species of *Ixodes*. Except for Diprotodontia, extant endemic orders carry a short tick fauna.

An interesting finding is that, even with such a lack of connectivity, the Australasian network is robust to disturbances because the stages of ticks display a low ontogenetic switch and persist on the same groups of hosts. The only tick with a relatively high phylogenetic diversity is *Ixodes holocyclus*, mainly in its adult stage. However, low ontogenetic switch is observed in the Australasian species, which is significant in four species out of 21 (*I. holocyclus*, *Ixodes cordifer*, *Ixodes granulatus* and *Ixodes tasmani*). However, looking at the alluvial chart in Fig. 9, the change in hosts among the three feeding stages is obvious. The most plausible explanation is that, even if such a leap among hosts occurs, it is produced among closely related taxa. The main partners of these ticks are Rodentia, Diprotodontia, Passeriformes and Dasyuromorphia for larvae. This scheme stands for nymphs, with Dasyuromorphia almost neglected as host for nymphs. Most of the species of ticks in this realm use Rodentia, Diprotodontia and Passeriformes as hosts for adults. There is a relatively higher degree of conservatism among Rodentia, Diprotodontia and Passeriformes, with most species recorded on one of these orders in any of their stages.

#### Afrotropical region

Results regarding the Afrotropical network of *Ixodes* spp. and their hosts are noteworthy (Figs. 10, 11). Such a network has a high modularity, with species/stages of ticks tightly linked to well-defined groups of vertebrates and centred around Carnivora, Artiodactyla and Rodentia, with high representation of Passeriformes, Afrosoricida and Eulipotyphla. The tick parasites of Chiroptera could constitute an independent cluster of the core of the network, but *I. simplex* (a typical parasite of bats) has also been recorded on Rodentia (as in other regions), linking the module of ticks + bats with the rest of the modules. Whether this constitutes a serendipitous finding or a consistent association remains to be determined.

Most *Ixodes* spp. recorded in the Afrotropics have a low phylogenetic diversity. Together with the low nestedness these results reveal a set of species tightly attached to their hosts, with a prominent disparity of families to which species of ticks may be linked. The low ontogenetic diversity recorded for the ticks of the genus in this realm is also significant. It seems that most of these results may be derived from an insufficient survey of the region and reflect only a partial representation of tick/host association.

## Discussion

Host specificity in ticks results from various factors including ecological, phylogenetic and physiological aspects [6]. Ticks exhibit different strategies; some species exhibit host conservationism (co-phylogenetic evolution), while others have been found on co-occurring vertebrates probably because both parasites and hosts exploit the same abiotic niche [4, 5, 17, 19]. The former would be a case of phylogenetic tracking in response to vertebrate speciation. Nevertheless, considering that ticks track the niche feeding on accessible hosts, the notion relies on the correlation between adaptation to the niche, which results in reciprocal selection of both ticks and hosts (coevolution without co-speciation [32]). Some studies have aimed to demonstrate that there is a direct relationship between the Major Histocompatibility Complex (MHC) and the structure of parasitic networks [33], an extreme not addressed in our study, but supporting the use of phylogenetic diversity in our proof-of-concept. The MHC is a large locus on vertebrate DNA containing a set of genes that code for proteins essential for the adaptive immune system. The structure of the network tested in [34] displayed a high nestedness. Since we did not explicitly test these interesting findings, we consider this to be an open door for future research, since the NODF values of our networks are very similar to those displaying relationships with MHC [33]. In any case, previous studies on tick-host networks demonstrated that host diversity increases the robustness and stabilizes the community, promoting the circulation of tick-borne pathogens that would otherwise be restricted to a few competent reservoirs, and probably have a lower efficiency of transmission [34–36].

The main background of host associations in *Ixodes* spp. is related to Aves (mainly Passeriformes), Rodentia, Carnivora and Artiodactyla. However, probable phylogenetic associations can be observed in several species of the subgenus *Eschatocephalus* that exploit bats in different biogeographical realms [37, 38]. The main network indicates that the major link of the complete network of ticks with bats is observed throughout the subgenus *Pholeoixodes*, a set of ticks that are parasites of vertebrates living in shelters [39]. A study demonstrated that the subgenus *Pholeoixodes* is not monophyletic [40], supporting previous results on the topic using *16S rRNA* and *cox1* genes [39]. The species in *Pholeoixodes* were grouped into two different clades that did not have a well-supported common node defining monophyly. It is interesting to note that the subgenus *Pholeixodes* currently includes ticks recorded on Carnivora or Aves. Therefore, it seems that these associations are ecological and evolved several times within a lineage of *Ixodes* and vertebrates living in burrows or caves and then separating into different “branches” that spread over different realms.

These results are not free of gaps, since they are based on data obtained from the literature; medically prominent species could be over-represented. An extra bias may come from the collection pressure on some hosts, which may be either abundant or easily trappable. These hosts and their ticks would be over-represented in comparison with protected or rare species. Adhering to previous studies [6] such observed high values of “specialists” are derived of the number of hosts surveyed and may therefore indicate a poor sampling of other putative hosts. The ticks with higher phylogenetic diversity are those most surveyed like the *I. ricinus* group of species, well known because its implications in human and animal health, and therefore widely surveyed. Other taxa are confirmed to be a complex of species and are distributed worldwide (i.e. *I. auritulus*) or a complex of species that is present in wide areas of several biogeographical realms (i.e. *I. affinis*).

The tight linkage of some *Ixodes* spp. to seabirds (e.g. Procellariformes and Sulifomes) is unquestionable and is not geographically restricted; it includes Sphenisciformes, which are unrelated to Procellariforms [41] but share habitats and, to some extent, nesting habits. These *Ixodes* spp. exploit nesting colonial seabirds in the circumpolar regions of both hemispheres and are considered seabird generalists [42]. These birds use cliffs of sea islands for nesting, commonly in large colonies. The involved *Ixodes* are endophilic and live in the material of the nest, feeding mainly on chicks in the breeding season [43, 44]. Ticks spend their off-host life cycle in the substrate surrounding the host nesting area, often in aggregates of several hundred individuals [45]. *Ixodes uriae* and its pelagic bird hosts were the first tick system studied to test for host-associated population genetic structure. It has been hypothesized [46] that *I. uriae* may consist of local populations adapted to specific hosts; McCoy and her team demonstrated that throughout its global distribution, this tick has indeed formed host-specific races independently evolving in different isolated regions [47–50].

Contemporary work employing population genetic tools to examine host-associated population structure in several ticks supports the idea that host specialization may evolve in some ticks but does not imply co-speciation. For example, it has been stated that ticks “seem to follow a pattern of being global generalists but local specialists” [51]. The results presented in this study support that generalist ticks may have “local hosts” that result from the environmental filtering of the whole set of hosts reported for the tick. Such a local view may distort the wide scale used in this study. Observational data available for some ticks support this view. For example, the relative infestation prevalence of *I. scapularis*, the main vector of Lyme disease in the eastern US, on rodent and lizard hosts shifts from north (on rodents) to south (on lizards) [52, 53].

We found that the most robust networks resulted for both Nearctic and Palearctic species of *Ixodes*, with high values of nestedness, partner diversity, functional complementarity and resilience to extinction. Fisher’s alpha is also highest for the Palearctic and the Nearctic. Nevertheless, the comparison of the high values of Fisher’s alpha in the network of the Afrotropical region with other indices suggests that these results may be biased because of deficient sampling. Network indices for the Afrotropical network are suggestive of ticks parasitizing a wide range of vertebrate families, but also point to a deficient surveying in the region. Therefore, the results that indicate to a high specificity of Afrotropical *Ixodes* spp. are probably derived from the partial knowledge of tick-host associations because of incomplete sampling or surveys restricted to livestock. For instance, the host distribution does not limit the tick ranges in Africa, implicitly assuming that there is not a clear phylogenetic association of the tick fauna with vertebrates in the Afrotropical region [54]. Furthermore, it has been demonstrated that African ticks of the same genus tend to not share similar habitats, something that could be expected in taxa radiating from a common lineage [55]. This suggests that close lineages of ticks in the region use different portions of the abiotic gradient to avoid competition for host resources. This could be a case of ecological adaptation, as a strategy of close tick species to avoid competence for the host’s resources, and is compatible with the network-derived findings: a clear ontogenetic switch, a low phylogenetic diversity and the use of a wide range of families of vertebrates as hosts. The topic has never been revisited to the best of our knowledge.

The results for the Australasian region are noteworthy. There is evidence of two lineages in the genus *Ixodes* [56], including the Australian-New Guinea (the *Ixodes tasmani* group) and the rest of the species [57]. Several authors [56–59] have reported that morphological and molecular characters weakly support monophyly of the genus *Ixodes*. However, other evidence has established that the genus *Ixodes* is not monophyletic [10], with Australian and non-Australian lineages forming separate clades (note *I. uriae*, which is phylogenetically basal, is included in the Australian clade). Interestingly, the network of Australian *Ixodes* species and their hosts has the highest number of disconnected clusters and the lowest functional diversity and functional complementarity (together with the Afrotropical). The Australian network exhibits a lack of clustering as well as fewer branches and links, perhaps because complete branches of vertebrates and their ticks became extinct. These findings are compatible with an event, such as mass extinction of vertebrates, shaping poor connectivity and large modularity because of loss of connections. This possible host extinction event is also observed in the extreme ontogenetic switch that occurs among virtually all stages of Australian *Ixodes* spp. and every order of hosts. This suggests a need to re-adapt to new groups of hosts that were originally not commonly used by the primitive Australian *Ixodes*.

These results have gaps. The results are based on data obtained from the literature, and medically prominent species could be over-represented. An extra bias may come from the collection pressure on some hosts, which may be either abundant or easily trappable. These hosts and their ticks would be over-represented compared with protected or rare species. Adhering to previous studies [6], such observed high values of “specialists” are derived from the number of hosts surveyed and may therefore indicate poor sampling of other putative hosts. Ticks with higher phylogenetic diversity are those most surveyed, such as the *I. ricinus* group of species, which is well known because of its implications in human and animal health and is therefore widely surveyed. Other taxa are confirmed to be a complex of species and are distributed worldwide (i.e. *I. auritulus*) or a complex of species that is present in wide areas of several biogeographical realms (i.e. *I. affinis*). The methods used in this study may be of interest for the elucidation of routes of circulation of tick-borne pathogens, with ticks exploiting a high diversity of reservoirs promoting more solid dissemination of pathogens. These hypotheses should be tested at the regional scale, linking with recent findings on the importance of vertebrate communities.

Tick-host preferences have both ecological and epidemiological meanings. The ontogenetic switch is an important piece of information to address the original question of this study about an ecological or phylogenetic origin for the associations between *Ixodes* spp. and their hosts. We proposed in this study the measure of the variability of the phylogenetic diversity of vertebrates used as hosts by the different stages as a method to evaluate the magnitude of such host switching. For those species for which enough data exist, a large interstage variability of hosts seems to be the rule. We hypothesize that, other than monoxenous species, ticks use the vertebrates that (i) provide an adequate environment for survival and development (e.g. molting, oviposition), such as the burrow of a vertebrate, (ii) contribute to the spread of the population to avoid competence phenomena for hosts in a site, either on long range migratory birds or the medium range distance favored by Carnivora or Artiodactyla, or (iii) support a massive increase of the tick population, feeding on large animals that consequently provide a large blood meal. The results support that, except for some cases (i.e. *Ixodes neuquenensis*, reported so far only on Microbiotheria), the association of *Ixodes* with vertebrates could be mainly ecological, ticks tracking the optimal environmental niche exploiting the vertebrates providing the best resilience of the tick population. The high values of ontogenetic switch detected in this study are suggestive of “leaps” among the hosts that colonize the most suitable tick environmental niche.

## Conclusion

We consider that, other than for a few species, the phylogenetic spectrum of vertebrates is not the main driver of host selection by *Ixodes* spp. The results also demonstrate that, in species for which enough data exist, the ontogenetic switch of hosts is evident. This fact suggests that *Ixodes* spp. cope with the environmental variability exploiting hosts that optimize the survival of the parasite under a gradient of abiotic conditions as the main selective pressure. We consider hosts as a secondary adaptation while sharing habitat with the ticks, with few exceptions. A strict phylogenetic link among vertebrates and ticks would exclude the shaping forces of the environment, restricting the ticks to the areas in which preferred hosts exist. Our results on the genus *Ixodes* do not support such a hypothesis, except in a few instances. Ticks with a wide ecological plasticity would colonize large areas, since they could use phylogenetically unrelated clades of hosts to which they are associated because of niche overlap or habitat sharing. The methods used in this study may be of interest for the elucidation of routes of circulation of tick-borne pathogens. These hypotheses should be tested also at the regional scale, linking with recent findings on the importance of the communities of vertebrates.

## Supplementary Information

**Additional file 1.** Complete network file (in “.graphml” format) as used throughout this study and the file of families of hosts, in “Nexus” format, obtained from the “Open Tree of Life”.

**Additional file 2: Figure S1.** Representation of the complete network of Ixodes spp., families and orders of hosts, arranged using the Force Atlas 2 algorithm. Circles (nodes) are taxa. The lines (links) between pairs of nodes are relationships between ticks and hosts (i.e. a species of tick reported on a family of hosts). The width of the line is proportional to the number of co-occuring events, but the length results only from the algorithm producing the chart. Colors are clusters or groups of ticks and hosts that interact more frequently among them than with other taxa.

## Data Availability

The datasets supporting the conclusions of this article are included within the article (and its additional files) as Additional File 1.
