# Supplementary figures and images for: Worldwide host associations of the tick genus Ixodes suggest relationships based on environmental sharing rather than on co-phylogenetic events
Source: Parasit Vectors. 2023 Feb 21;16:75. doi: 10.1186/s13071-022-05641-9 (PMC9945728; doi:10.1186/s13071-022-05641-9)

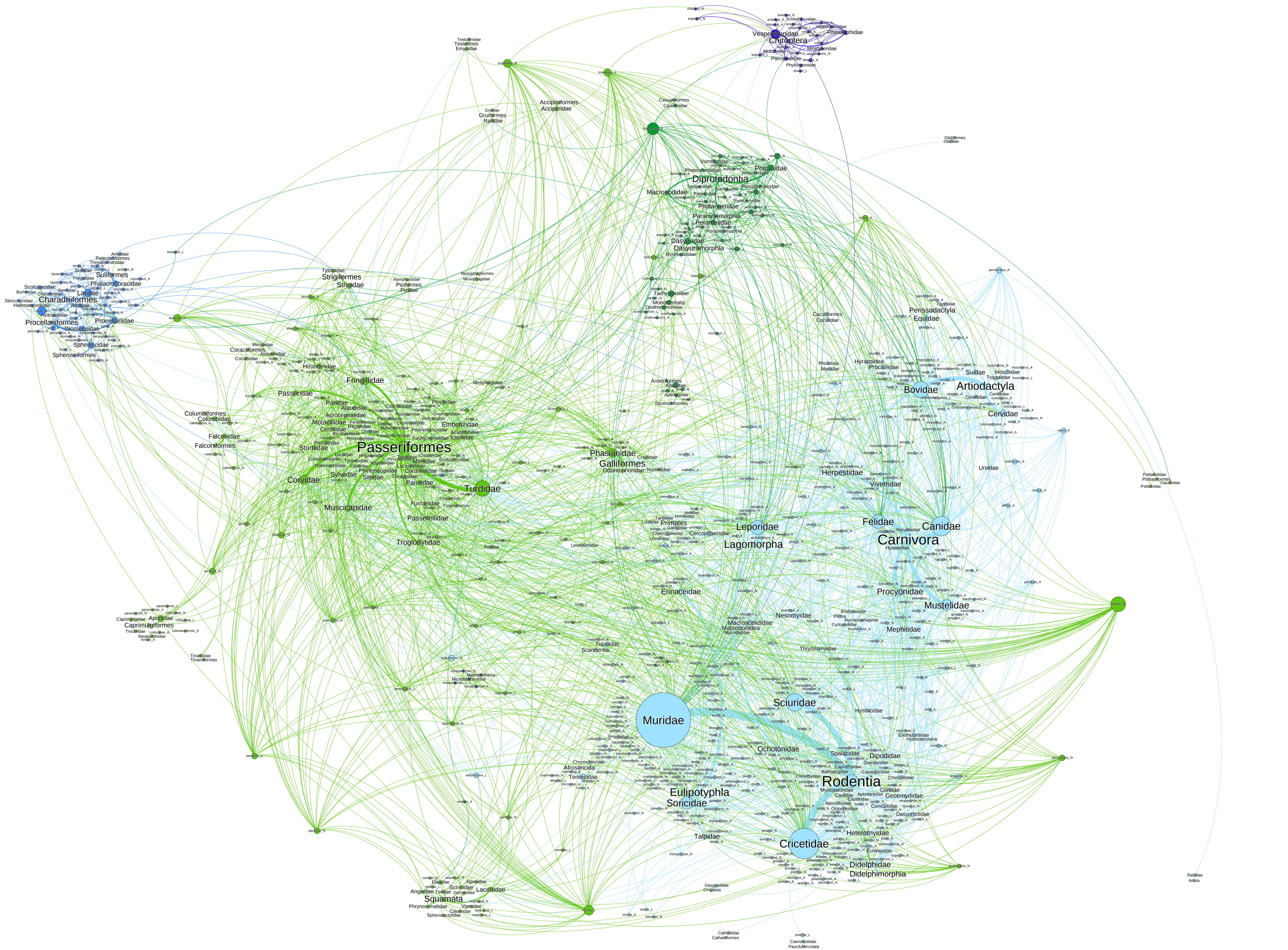

Supplement: Supplementary file 2 — Additional file 2: Figure S1. Representation of the complete network of Ixodes spp., families and orders of hosts, arranged using the Force Atlas 2 algorithm. Circles (nodes) are taxa. The lines (links) between pairs of nodes are relationships between ticks and hosts (i.e. a species of tick reported on a family of hosts). The width of the line is proportional to the number of co-occuring events, but the length results only from the algorithm producing the chart. Colors are clusters or groups of ticks and hosts that interact more frequently among them than with other taxa. [file 13071_2022_5641_MOESM2_ESM.pdf]
